# How paternalistic leadership influences teachers' resistance to STEM reform: the mediating role of teachers' STEM literacy and the moderating role of work engagement

**DOI:** 10.3389/fpsyg.2026.1726148

**Published:** 2026-02-23

**Authors:** Yao Zhang, Dongchen Zhao

**Affiliations:** College of Educational Science, Harbin Normal University, Harbin, Heilongjiang, China

**Keywords:** paternalistic leadership, STEM literacy, STEM resistance, teacher resistance, work engagement

## Abstract

**Introduction:**

Identifying the factors associated with teachers' resistance to STEM and understanding their interrelationships are crucial for advancing STEM education. This study examines the relationships between leadership styles and teacher resistance, with STEM literacy and work engagement serving as mediating and moderating variables, respectively.

**Methods:**

A survey was conducted among 1,221 primary and secondary school teachers from a provincial capital in northern China. Structural equation modeling was used to examine the relationships among leadership styles, teachers' STEM literacy, work engagement, and resistance to STEM reform.

**Results:**

The results indicate that: Authoritarian leadership was significantly and positively related to teachers' STEM resistance, whereas benevolent-moral leadership had no direct statistical effect. However, the relationship between leadership style and resistance became more complex when teachers' STEM literacy was taken into account. Authoritarian leadership was indirectly associated with teachers' STEM resistance via STEM literacy, whereas the association between benevolent-moral leadership and resistance was fully mediated by STEM literacy. Work engagement was negatively related to teachers' STEM resistance. Furthermore, work engagement moderated the relationship between leadership style and STEM literacy. The positive relationship between benevolent-moral leadership and STEM literacy was stronger at higher levels of work engagement, whereas the association for authoritarian leadership was positive only at low levels of engagement and turned negative as work engagement increased.

**Discussion:**

Based on these findings, enhancing teachers' STEM literacy and willingness to participate in STEM education requires promoting a culture of benevolent-moral leadership while carefully defining the boundaries of authoritarian management practices. Meanwhile, differentiated incentive and support mechanisms should be developed according to teachers' levels of work engagement, thereby fostering sustained professional development and active involvement in STEM education reform.

## 1 Introduction

As a core element driving innovation capacity and economic competitiveness, STEM education has become a strategic focus of global educational reform. STEM refers to an interdisciplinary approach to learning that integrates Science, Technology, Engineering, and Mathematics and emphasizes problem-solving, critical thinking, design, and innovation (Hasanah, 2020; Sheth and Pathak, 2023; Siekmann, 2016). Countries worldwide have incorporated STEM education into their national curricula and are striving to advance STEM course practices. As the primary implementers of curriculum reform, teachers play a key role in policy implementation, and their professional literacy and reform willingness to implement reform are closely related to implementation outcomes. However, research has shown that STEM education reform is facing teacher resistance (Al Salami et al., 2017; Masry-Herzalah and Dor-Haim, 2022; Rodriguez, 1998; Spector et al., 2007) due to various constraints such as institutional factors, resource limitations, and teachers' professional capabilities (Escudeiro et al., 2024; Huang and Asghar, 2018; Oreg, 2018). This resistance behavior leads to a decline in policy implementation effectiveness, traps STEM education in formalism, undermines the development of students' core competencies and runs counter to the national strategy of cultivating innovative talents. In this context, identifying the root causes and interaction mechanisms of teachers' resistance to STEM is crucial for advancing curriculum implementation and enhancing the quality of STEM education.

Research on teacher resistance in the field of education has yielded relatively rich findings (Huddleston et al., 2024; Poole, 1991), but research on teacher resistance in STEM education has not yet received sufficient attention, especially lacking empirical research on the causes and internal mechanisms of teachers' resistance to STEM. Previous studies have revealed the complex causes of teacher resistance (e.g., Ballet and Kelchtermans, 2009; Han, 2011; Hargreaves, 2005; Rong, 2007; Wang, 2016; Wang et al., 2007; Yin, 2015), which can be broadly categorized into internal factors and external factors related to the surrounding environment. Internal factors mainly focus on professional literacy and emotions, while external factors are mostly related to organizational management practices. In the context of STEM education practices in schools, these factors highlight three teacher-related dimensions: professional literacy, which concerns teachers' ability to effectively implement STEM courses; emotional commitment, reflected in their work engagement, that is, whether they are willing to do well; and management practices, which involve organizational conditions such as leadership and institutional support—namely, how teachers are expected, required, and supported to act in implementing STEM education. Given this, exploring the relationships among teachers' STEM literacy, work engagement, and school management practices, as well as their mechanisms of influence on teachers' STEM resistance, will help reveal the underlying causes and critical pathways of teacher resistance to STEM education. Internal causes are the basis of a thing's development, while external causes are the conditions and exert their influence through internal causes. This study takes school management practices as the main external factor and further focuses on the leadership style within the teachers' organizations. It considers teachers' STEM literacy and work engagement as the main internal factors, exploring how leadership styles influence teachers' STEM resistance through teachers' STEM literacy and work engagement, and clarifying the underlying mechanisms linking the four variables. The results of this study can provide empirical evidence and strategic insights for improving the management effectiveness of STEM education and alleviating teachers' STEM resistance, thereby promoting the long-term and substantive development of STEM education.

## 2 Literature review

### 2.1 Teachers' agency and resistance to STEM

Curriculum implementation was a dynamic organizational process shaped over time through interactions among project goals, methods, and the institutional setting (McLaughlin, 1976). Reform outcomes hinge on how teachers engage with and enact change during implementation (Fullan, 2007), making teacher agency central to this process. Teacher agency refers to educators' capacity to exercise purposeful professional judgment and take action within structural and cultural conditions, thereby influencing both their work and professional environments (Priestley et al., 2015). In their ecological model of teacher agency, Priestley et al. (2015) argue that agency is not a fixed individual trait but emerges and is continually shaped through iterative interactions among three interrelated dimensions: teachers' life-course experiences, professional beliefs, and accessible resources (the individual dimension); school organizational structures, management culture, and accountability arrangements (the structural dimension); and broader policy discourses, professional norms, and social expectations (the cultural dimension).

In reform contexts, agency may be expressed through active adaptation, innovation, and leadership, but it may also take the form of resistance when teachers perceive a disconnect between reform expectations and their professional values, practical knowledge, or students' needs (Biesta et al., 2017; Achinstein and Ogawa, 2006; Huddleston et al., 2024). Research on teacher agency and reform further indicates that teachers' interpretations of institutional logics and reform messages can lead to divergent pathways—adaptation, negotiation, or opposition—thereby shaping whether instructional practices change or remain stable (Bridwell-Mitchell and Sherer, 2017). Within STEM-related reforms, empirical studies demonstrate that teacher agency is crucial for initiating and sustaining curricular change, suggesting that teachers' competence and motivation jointly determine whether reforms are enacted creatively or met with passive resistance (Balgopal, 2020).

Resistance, defined as a negative personal orientation toward the notion of change, is generally viewed as an obstacle on the way to effective adaptation and improvement (Oreg, 2018). Teacher resistance is a cognitive or behavioral reaction where teachers may utilize their agency to resist educational reform when the anticipated reform conflicts with their long-standing institutional beliefs and practices (Bridwell-Mitchell and Sherer, 2017). As early as 1977, Oliver pointed out that curriculum reform encountered some obstacles from teachers (Oliver, 1977, p. 327). Resistance, as a way for teachers to cope with reform, manifests in complex and diverse forms. Hargreaves (2005) identified varied responses to change among later-career teachers, including continuing renewal, positive focusers, disenchanted, and negative focusers. Other studies further classified teacher resistance into explicit and implicit forms (Liu, 2008), individual or collective, formal or informal, and ranging in intensity from passive inaction to overt opposition (Wang, 2016). Based on teachers' understanding and acceptance, resistance can also be divided into wise acceptance, blind acceptance, wise resistance, and blind resistance (Xia, 2008). Together, these frameworks underscore the multidimensional and dynamic nature of teacher resistance in educational reform. Existing research has also revealed the reasons for teacher resistance, which can be summarized into personal and environmental aspects. Personal reasons mainly involve teachers' educational philosophies and value orientations (Han, 2011); their teaching knowledge, abilities, and professional experience (Wang et al., 2007; Song and Su, 2004); and a range of psychological or motivational constraints, such as limited energy and behavioral inertia (Rong, 2007), self-interest and emotional needs (Wang, 2016), autonomy in professional judgment, and anxiety about the uncertainty of reform (Han, 2011; Ballet and Kelchtermans, 2009; Benett, 1980; Kelchtermans, 2005). Environmental factors involve support resources such as teacher training, organizational culture and management, evaluation mechanisms and other work structural constraints (Yin, 2015; Ballet and Kelchtermans, 2009; Kelchtermans, 2005). Deeper cultural conflict factors include examination culture, academic success culture, curriculum culture, and other diverse cultural systems. These factors interact to shape teachers' complex responses to educational reform (Wan and Wang, 2005; Lu and Wen, 2005; Song et al., 2005).

### 2.2 Leadership style and teachers' resistance to STEM

Existing research indicates that organizational cultural atmosphere is an important factor influencing curriculum implementation (Fullan and Pomfret, 1977). Leadership style, embedded within organizational culture, is significantly associated with the effectiveness of curriculum implementation Hall and Hord, (2015, p. 133–159; Shieh, 1996).

Widely used international leadership frameworks provide additional lenses for understanding the dynamics between leadership and teacher resistance. Transformational leadership—by articulating a shared vision, offering individualized support, and stimulating teachers' intellectual engagement—has been linked to stronger teacher commitment and greater effort toward school reform (Geijsel et al., 2003), with meta-analytic evidence in educational settings also suggesting positive effects on teacher-related outcomes (Leithwood and Sun, 2012). In contrast, transactional leadership is conceptualized as an exchange process in which leaders clarify requirements and specify the conditions and rewards for meeting them, and such exchange-based influence may elicit compliance without necessarily cultivating internalized commitment and involvement, which often require going beyond transactional exchanges (Bass and Riggio, 2006, p. 4). Servant leadership, which centers on empowerment, humility, and follower development (van Dierendonck, 2011), has also been associated with more supportive climates that facilitate change readiness (Latif et al., 2024). (Hall and Hord 2015, p. 149–150) classified leadership styles into initiating, managing, and responding types, and found that teachers achieved the highest level of success under the management of leaders with an initiating style in curriculum implementation.

However, cross-cultural research indicates that culturally embedded values, such as expectations regarding authority and hierarchy, shape leader–follower relations (Hofstede, 2001). This underscores the need to examine leadership approaches that are culturally resonant in Confucian-influenced contexts. Paternalistic leadership—typically conceptualized as a combination of authoritarianism, benevolence, and morality—has been widely discussed as a culturally rooted and prevalent leadership pattern in Chinese organizations Farh and Cheng, (2000; Pellegrini and Scandura, 2008), with meta-analytic evidence further indicates its differential associations with follower outcomes across its dimensions (Bedi, 2020). In Chinese educational settings, where reform initiatives (including STEM reform) are often implemented through top-down governance mechanisms, paternalistic leadership provides a culturally grounded lens for understanding how leadership relates to teachers' resistance and reform-related behaviors. Paternalistic leadership is a unique leadership model that integrates three dimensions: authoritarianism, benevolence, and morality. Its theoretical construction originated from Silin's analysis of Chinese organizational management and was later formalized by (Cheng et al. 2000) as a tripartite framework. They defined paternalistic leadership as a leadership style that combines strict discipline and authority with paternal benevolence and morality and is rooted in an individualistic atmosphere (Farh and Cheng, 2000). As a composite leadership model, the ternary structure of paternalistic leadership (authoritarianism, benevolence, morality) influences the work behaviors of organizational members. Teacher resistance is, at its core, a response to educational authoritarianism (Parra-Perez et al., 2022), which enforces obedience through hierarchical authority and strict control (Cheng et al., 2000). This “strict father” style of management may be perceived by teachers as a threat to their professional autonomy, thereby triggering resistance. Existing research has confirmed that administrative command-style teaching reforms or standardized accountability policies can evoke negative or resistant attitudes by depriving teachers of professional autonomy (Sera-Sirven, 2021; Terhart, 2013). Hall and McGinity (2015), through qualitative research, revealed that authoritative leadership makes teacher resistance implicit through coercion, the creation of fear, and false empowerment.

In contrast, the other two dimensions of paternalistic leadership present a different picture. Benevolent leadership, through long-term emotional investment and individualized care, easily establishes a moral responsibility relationship based on “gratitude and reciprocation” (Cheng et al., 2000). This relationship is reflected in organizational behavior as the leader's protection and care for subordinates, which, in turn, elicits obedience and loyalty as forms of reciprocal response—a psychological contract fundamentally distinct from the social exchange frameworks emphasized in Western leadership theories. Oreg's (2006) research also provides theoretical support for this, as his research found that leaders' supportive behaviors can alleviate teachers' anxiety about the uncertainty of reform and thus reduce defensive resistance tendencies. The influence of moral leadership is even more profound. When leaders establish professional authority through moral demonstration, they can inspire teachers' enthusiasm (Zheng et al., 2020) and prompt teachers to internalize reform goals through moral demonstration and value consistency (Pellegrini and Scandura, 2008), thereby reducing resistance. Based on the above theories, this study proposes the following research question:

**H1:** Authoritarian leadership positively predicts teachers' STEM resistance.
**H2:** Benevolent and moral leadership negatively predict teachers' STEM resistance.

### 2.3 Paternalistic leadership and teacher literacy

Paternalistic leadership theory is rooted in the Confucian cultural context, and its core lies in the traditional management concept of combining favors and discipline. Leaders influence the attitudes and behaviors of organizational members through moral role modeling, benevolent care, and authoritarian control (Cheng et al., 2000). Compared with Western transformational leadership, which supports and motivates employees, and transactional leadership, which forms contracts with employees regarding goals and expected outcomes (Caniëls et al., 2018), paternalistic leadership is characterized by the interaction of ethical authority and emotional attachment in the workplace (Pellegrini and Scandura, 2008). This contradiction may play a different role in STEM education reform than in traditional teaching scenarios. The uniqueness of the teacher literacy required for STEM courses lies in its high dependence on interdisciplinary integration ability, technical practice ability, and innovative teaching strategies (Margot and Kettler, 2019). Unlike traditional subject teachers, STEM teachers are required to integrate scientific inquiry, engineering design, and other complex competencies into authentic, real-world problem-solving tasks (National Research Council, 2012, p. 7–22; Song and Guan, 2022). However, teaching transformation faces multiple resistances: on the one hand, teachers need to cope with the dual pressures of knowledge renewal and curriculum development; on the other hand, resource shortages and the misalignment of assessment systems (such as emphasizing standardized tests rather than innovation ability) exacerbate occupational burnout (Margot and Kettler, 2019; Dong, 2016). Therefore, STEM professional literacy requires not only sufficient space for teachers' autonomous exploration and the exercise of professional agency, but also clear organizational support and guidance. The balance between these two dimensions may lead to markedly different outcomes, depending on how the dual nature of paternalistic leadership is enacted.

Existing research has confirmed a significant correlation between leadership style and teacher literacy (Hasti and Purwanto, 2024; Iba et al., 2023; Chang, 2012; Cai et al., 2024). For example, transformational leadership significantly improves teachers' digital literacy (Susanti et al., 2023), and technological leadership helps enhance teachers' technological literacy and technology integration ability (Chang, 2012). In the context of Chinese culture, Cai et al. (2024) found that benevolent and moral leadership positively predict the establishment of teacher professional learning communities, while authoritarian leadership has no significant impact.

The Conservation of Resources Theory (Hobfoll, 1989) can provide theoretical support for the influence of leadership on teacher literacy. This theory emphasizes that individuals cope with stress by acquiring and protecting valuable resources. In educational contexts, leaders convey various resources through their behavioral styles, including not only material resources such as skill training and technical equipment but also social resources such as organizational trust and psychological safety (Hobfoll et al., 2018; Cai et al., 2024). The acquisition of these resources can reduce teachers' sense of exhaustion when facing reform pressures and enhance their professional confidence, thereby contributing to the development of STEM literacy.

Although existing research has revealed the general correlation between leadership style and teacher literacy, there are two limitations. First, most of the literature focuses on Western transformational leadership theories and lacks discussion on the cultural applicability of paternalistic leadership in Chinese society. Second, the uniqueness of teachers' STEM literacy (such as interdisciplinary integration ability, technical practice ability, and innovative teaching strategies) has not been fully incorporated into the research framework of teacher literacy. Therefore, this study proposes:

**H3:** Authoritarian leadership negatively predicts teachers' STEM literacy.
**H4:** Benevolent and moral leadership positively predict teachers' STEM literacy.

### 2.4 Teacher STEM literacy and teachers' resistance to STEM

The relationship between teacher literacy and teacher resistance can be traced back to the core propositions of educational reform theories. Fullan and Pomfret (1977) pointed out that if teachers believe that a curriculum is overly complex and requires the adoption of entirely new teaching strategies, their willingness to implement the reform—as well as the extent to which they do so—is significantly diminished. This argument is echoed by Hall's research, which suggests that teachers' attitudes, abilities, and values are important factors affecting curriculum implementation (Hall and Wu, 2004, p. 167–173). Existing research has found that in curriculum reform, factors such as insufficient personal abilities and experience (Zaltman and Duncan, 1977, p. 21–22), teachers' technological literacy gaps (Sera-Sirven, 2021), and perceptions of the uncertainty and risks of reform (Howard, 2013) are important causes of teacher resistance. Teachers' educational philosophies are significantly related to teacher resistance. Teachers with traditional educational orientations (perennialism and essentialism) are positively correlated with teacher resistance, while teachers with contemporary educational orientations (progressivism and reconstructionism) are negatively correlated with teaching resistance (Alanoglu et al., 2022). Additional research from the perspective of teacher training has demonstrated that professional development support fosters teachers' initiative in reform efforts, thereby mitigating their resistance to change (Howard, 2013; Pei and Yang, 2019; Sannino, 2010; Sera-Sirven, 2021).

In summary, it can be inferred that teachers' professional literacy in the STEM field affects their acceptance of reform measures and new teaching strategies. Higher STEM literacy will help teachers better adapt to the challenges and changes brought about by educational reform, thereby reducing their sense of maladjustment and resistance to reform. Therefore, this study proposes:

H5: Teacher STEM literacy negatively predicts teachers' resistance to STEM.

### 2.5 The mediating role of teacher STEM literacy

Drawing on the arguments in Sections 2.3 and 2.4, this study posits that teachers' STEM literacy mediates the relationship between paternalistic leadership and teachers' resistance to STEM reform. This proposition is theoretically grounded in a coherent logic chain: leadership style is associated with teachers' STEM literacy, which in turn is related to the extent of their resistance to the reform.

First, as discussed in Section 2.3, paternalistic leadership is a key antecedent of teachers' STEM literacy. According to Conservation of Resources theory, individuals cope with stress by acquiring and protecting valued resources. In educational settings, leaders convey multiple forms of resources through their behavioral styles, including material resources (e.g., skills training and technological equipment) and social resources (e.g., organizational trust and psychological safety) (Hobfoll et al., 2018; Cai et al., 2024). Access to these resources can reduce teachers' feelings of exhaustion when facing reform-related pressures, strengthen their professional confidence, and thereby facilitate the development of STEM literacy. In particular, psychological safety has been shown to encourage members to speak up about errors and to try new approaches, thereby promoting learning behaviors within teams (Edmondson, 1999). By contrast, authoritarian leadership is more likely to trigger resource depletion and draining psychological processes (e.g., rumination and emotional exhaustion) by intensifying control and constraining autonomy (Zhang et al., 2022), which undermines teachers' sustained learning and competence development.

Second, as argued in Section 2.4, teachers' STEM literacy serves as a proximal determinant of their resistance to reform. Educational change research suggests that when teachers perceive reform demands as overly complex, entailing higher levels of risk, and requiring entirely new strategies, their willingness to implement the reform—as well as their actual investment—declines substantially (Fullan and Pomfret, 1977; Hall and Wu, 2004). Research from the perspective of teacher training likewise indicates that support for professional development can activate teachers' agency for reform and, in turn, reduce their resistance (Howard, 2013; Sera-Sirven, 2021).

Taken together, these two pathways suggest that leadership styles predict teachers' STEM literacy for coping with reform, which in turn is associated with their attitudinal and behavioral responses to high-demand change. This mediation logic further aligns with a classic model of teacher change: professional development supported by external resources and internal motivation often leads to changes in practice, and teachers' attitudes and beliefs begin to shift only after the effects of these changes are experienced and validated (Guskey, 2002).

In line with this reasoning, the following hypotheses are advanced:

**H6:** Teacher STEM literacy mediates the relationship between authoritarian leadership and teachers' STEM resistance.
**H7:** Teacher STEM literacy mediates the relationship between moral/benevolent leadership and teachers' STEM resistance.

### 2.6 Work engagement and teachers' resistance to STEM

As one of the earliest studies on resistance to change, (Coch and French 1948) pointed out that employee participation has a positive impact on reducing employee resistance to change. Building on this foundation, later studies have since turned their attention to strategies for facilitating change by strengthening employees' readiness and willingness to engage in the process (Armenakis et al., 1993; Piderit, 2000). In this context, work engagement, as an individual's degree of emotional, cognitive, and behavioral involvement in work, has become an important factor for scholars to explore employees' willingness to change. Matthysen and Harris's (2018) research shows that high work engagement can improve employees' readiness for change, that is, employees with high work engagement are more likely to adapt to and support change. van den Heuvel et al.'s (2010) Personal Resource Adaptation Model further suggests that work engagement, personal resources, willingness to change, change strategies, and the work environment collectively form a gain cycle. Within this cycle, willingness to change and the use of change strategies positively influence work engagement, while work engagement, in turn, enhances willingness to change through the mediating effect of personal resources. Armenakis et al. (1993) also identified employees' active engagement as the core component of the Building Readiness for Change model. They argued that employees' awareness of both the necessity and feasibility of change—that is, their willingness and readiness—plays a critical role in facilitating successful change implementation.

Although prior research has seldom explored the link between work engagement and resistance to change in educational contexts, theoretical insights suggest that engagement—reflecting focus and dedication—can reduce resistance by increasing teachers' readiness for change. Highly engaged teachers are more willing to invest personal resources such as psychological capital and self-efficacy, which strengthens their identification with reform and lowers resistance rooted in cognitive bias. Therefore, it is hypothesized that:

**H8:** Work engagement negatively predicts teachers' STEM resistance.

### 2.7 Work engagement as a moderating variable

Work engagement is a positive and fulfilling work-related mental state that includes three core dimensions: vigor, dedication, and absorption (Schaufeli et al., 2002). As a representation of an individual's emotional, cognitive, and behavioral involvement in work, work engagement has often been regarded as a key psychological mechanism connecting external resources and individual work performance in previous research (Bakker and Demerouti, 2007).

Research indicates that employees with high levels of work engagement are more likely to achieve task goals and produce better performance (Kahn, 1992). Specifically for teachers, their level of work engagement is positively correlated with creativity (Desianti et al., 2023), digital ability (Sang et al., 2023), and professionalism (Jeong et al., 2016; Sang et al., 2023).

From the perspective of influencing factors, the formation of work engagement is influenced by the combined effects of internal and external resources (Kahn, 1992). Leadership, as an important external resource, has a significant impact on employees' work engagement by shaping a psychologically safe environment and providing supportive resources (Breevaart et al., 2014; Caniëls et al., 2018; Tims et al., 2011). Research in the field of education confirms that leaders who provide necessary support to teachers can enhance teachers' work motivation (Deluma et al., 2020; Iba et al., 2023) and increase their work engagement (Rahmadani et al., 2024).

Although most research regards professional literacy as an antecedent variable of work engagement, existing research has shown that there is a bidirectional positive correlation between the two. Hontake and Ariyoshi's (2016) research proves that the relationship between work engagement and professional literacy conforms to the Job Demands-Resources Model, that is, work engagement and professional literacy can form a “gain spiral”: individuals stimulate resource acquisition through work engagement, thereby improving their work ability, and higher ability in turn promotes deeper engagement. At the same time, Isah et al. (2021) found that work engagement plays a mediating role in the process by which emotional commitment affects employees' abilities, indicating that it can promote ability development. In the field of education, Galleon and De Leon (2023) pointed out that the work engagement of primary school teachers significantly improves their academic ability. It can be seen that work engagement is not only an external manifestation of emotional drive and motivation but also a psychological mechanism that prompts individuals to continuously learn, accumulate experience, and improve their abilities. From a dynamic development perspective, sustained high-level work engagement can build an individual's ability foundation, strengthen their professional competence, and continuously expand their work resources and development space through a positive cycle.

The Conservation of Resources Theory (Hobfoll et al., 2018) further provides theoretical support for the moderating role of work engagement. This theory suggests that individuals obtain more resources through resource investment, and those with richer resource reserves are more capable of achieving further resource acquisition. Work engagement is described as a state of abundant energy resources, which originates from a continuous process of resource accumulation (Gorgievski and Hobfoll, 2008). Therefore, employees who invest more emotional, cognitive, and physical resources in their work (high-engagement employees) may be more capable than low-engagement employees of achieving resource gains through leadership and thus improving their professional literacy. Conversely, even if leaders provide a certain degree of external resources, employees lacking engagement may find it difficult to benefit from them (Johnson and Jiang, 2017). This differentiated resource conversion ability makes work engagement a potential boundary condition for regulating the relationship between leadership behavior and teachers' professional development. Therefore, this study proposes:

**H9:** Work engagement negatively moderates the relationship between authoritarian leadership and teachers' STEM literacy.
**H10:** Work engagement positively moderates the relationship between moral/benevolent leadership and teachers' STEM literacy.

**Figure 1:**
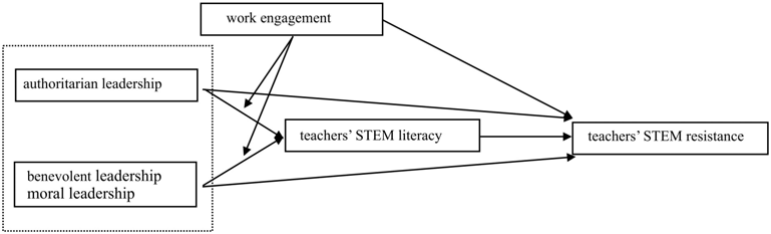
Theoretical hypothesis model.

Based on the above analysis, this study constructs a theoretical hypothesis model as shown in Figure 1.

## 3 Methods

### 3.1 Participants

This study targeted primary and secondary school teachers of STEM-related disciplines (including science, mathematics, information technology, physics, chemistry, biology, and geography) in Harbin, a provincial capital located in northern China. Stratified cluster sampling was employed to select sample schools from both the main urban area and surrounding counties and towns of Harbin. With the assistance of educational research institutions, an invitation to participate in the survey was extended to teachers in the sample schools, and an online questionnaire was distributed for voluntary completion. A total of 1,998 questionnaires were collected. Invalid questionnaires filled out carelessly were excluded based on three criteria: (1) excessively short response times; (2) overly consistent answers; and (3) contradictory responses to lie-detection questions. Ultimately, 1,221 valid questionnaires were obtained, representing an effective response rate of 61%. Among the valid respondents, there were 260 male teachers and 961 female teachers; 655 primary school teachers and 566 junior high school teachers; and 801 teachers from the main urban area and 420 teachers from counties and towns.

### 3.2 Research instruments

#### 3.2.1 Teachers' resistance to STEM scale

Most prior studies assessing teacher resistance have relied on the *Employee Resistance to Change Scale* developed by Oreg (2006). To enhance the scale's applicability to the STEM education context, this study revised the item wording while retaining the original scale's cognitive, affective, and behavioral dimensions. For instance, the original item “I was stressed by the change” was modified to “Implementing STEM courses makes me feel stressed.” The revised scale consisted of 11 items rated on a five-point Likert scale, ranging from “very inconsistent” (1) to “very consistent” (5). Higher scores indicated greater resistance to STEM course implementation among teachers. The internal consistency test revealed a Cronbach's α coefficient of 0.878, indicating good reliability of the questionnaire. Exploratory factor analysis (EFA) was conducted to examine the construct validity of the scale, yielding a Kaiser-Meyer-Olkin (KMO) measure of sampling adequacy of 0.863 (*p* < 0.001). Standardized factor loadings for the items ranged from 0.714 to 0.906, demonstrating that the items effectively reflected the information of their respective dimensions. The structural equation model demonstrated a good fit to the data, with the following fit indices: χ^2^/df = 2.937, RMSEA = 0.044, CFI = 0.992, GFI = 0.992, and TLI = 0.989.

#### 3.2.2 Paternalistic Leadership Scale

The Paternalistic Leadership Scale developed by Chen et al. (2015) was used to assess teachers' perceptions of leadership styles in their schools. This scale is a simplified version of the Paternalistic Leadership Scale developed by Cheng et al. (2000), encompassing three dimensions: benevolence, morality, and authoritarianism, with a total of 18 items. A five-point Likert scale was used for scoring, ranging from “very inconsistent” to “very consistent”, with scores from 1 to 5. Each dimension was scored independently, with higher scores indicating a stronger tendency toward a specific leadership style.

Based on survey data, we conducted an confirmatory factor analysis (CFA) on the 18-item Paternalistic Leadership Scale. The results indicated that the conventional three-factor model (benevolence, morality, and authoritarianism) fit the data poorly (χ^2^/df = 13.983, RMSEA = 0.103, CFI = 0.933, TLI = 0.923, GFI = 0.845), and there was a high correlation between benevolent leadership and moral leadership (*r* = 0.901, *p* < 0.001). In contrast, a two-factor model that combined benevolence and morality into a single dimension (benevolence–morality), alongside authoritarianism, showed a substantial improvement in fit (χ^2^/df = 7.265, RMSEA = 0.072, CFI = 0.985, TLI = 0.979, GFI = 0.962), suggesting a better balance between fit and parsimony. Information criteria further favored the two-factor model (AIC = 1,756.562, BIC = 1,927.494) over the three-factor model (AIC = 1,923.725, BIC = 2,122.914). These results suggest that benevolence and morality may be difficult to distinguish empirically in the sample of this study. Therefore, we re-examined from both theoretical and empirical perspectives whether benevolence and morality should be combined into a single factor.

The tripartite model of paternalistic leadership (benevolence, morality, and authoritarianism) was adapted from an earlier bipartite model comprising “benevolence” and “authority” (Cheng, 1995). Cheng et al. (2000) revised this framework by separating “morality” from “benevolence” in order to more clearly distinguish the ethical dimension from the affective component. However, their findings also revealed a very high correlation between benevolence and morality (r = 0.88), suggesting substantial conceptual overlap between the two. Later studies have also challenged the empirical separability of morality from benevolence. In East Asian settings, benevolence and morality often co-occur, because affective care and moral exemplification are typically enacted together (Farh and Cheng, 2000). Accordingly, Chan et al. (2013) raised concerns about the cross-contextual generalizability and discriminant validity of the morality dimension.

From a broader theoretical perspective, paternalistic leadership has been widely conceptualized around a control–care duality, whereby benevolence denotes a relationship-oriented stance grounded in leaders' concern for subordinates and a sense of responsibility and obligation toward them (Aycan, 2006). Typological research based on this dual framework further classifies paternalistic leadership into benevolence-dominant, authoritarian-dominant, and classical types (refers to the salient combination of both leadership components), in which benevolence and morality typically appear together as joint components of a positive, relationship-oriented leadership pattern rather than as functionally independent elements (Wang et al., 2018). Hiller et al. (2019) explicitly argued that morally grounded benevolence and authority capture the core and essence of paternalistic leadership, and that morality does not need to be retained as a separate dimension, recommending that future leadership research discontinue the independent use of a morality dimension.

Driven by the above theories, we refined the 18-item Paternalistic Leadership Scale to ensure model parsimony and stable parameter estimation in subsequent structural equation modeling analyses. We conducted an Exploratory Factor Analysis (EFA) to examine the dimensional structure of the paternalistic leadership scale. After rotation and remove the items with low factor loadings or high residual covariances, a two-factor structure with 9 items emerged: benevolent and moral leadership items loaded onto the first factor, while authoritarian leadership items comprised the second. Factor loadings for each item ranged from 0.714 to 0.906. Subsequently, CFA was conducted on the refined scale. The CFA results showed that the scale with two-factor had good fit (χ^2^/df = 3.040, RMSEA = 0.041, CFI = 0.996, TLI = 0.994, GFI = 0.988), which is better than the original scale with three factors and 18 items.

The final scale also showed moderate internal consistency, as indicated by a Cronbach's α of 0.633. The KMO measure of sampling adequacy was 0.951 (*p* = 0.000). The composite reliability (CR) values were 0.950 for the “benevolence–morality” dimension and 0.926 for “authoritarianism”, both exceeding the recommended threshold of 0.7. The average variance extracted (AVE) was 0.827 and 0.757, respectively, indicating adequate convergent validity and good internal consistency of the scale (see Table 1).

**Table 1:** Average variance extracted (AVE) and inter-construct correlation coefficients for paternalistic leadership.

**Constructs**	**AVE**	**Authoritarian leadership**	**Benevolence-moral leadership**
Authoritarian leadership	0.757	**0.870**	
Benevolence-moral leadership	0.827	−0.427	**0.909**

Bold values on the diagonal indicate the square roots of AVE for each construct; off-diagonal values are inter-construct correlations.

#### 3.2.3 Teacher STEM literacy scale

Teachers' STEM literacy was assessed using a scale specifically developed for this study. The scale was grounded in the dimensions and indicators outlined in the *STEM Teacher Competency Standards (Trial Edition)*, a national framework issued by the National Institute of Education Sciences Center for STEM Education (2018). The scale consisted of four dimensions: value orientation toward STEM Education; disciplinary foundations and interdisciplinary integration; development and implementation of STEM curriculum; and curriculum evaluation. A five-point Likert scale was used for scoring, ranging from “very inconsistent” to “very consistent”, with scores from 1 to 5. Higher scores reflected higher levels of self-perceived STEM literacy among teachers.

After an initial pool of 31 items was generated, six experts (two professors in STEM education, two experienced STEM teachers, and two specialists in measurement and evaluation) were invited to independently and anonymously review the items. The experts rated each item in terms of content relevance, representativeness, and clarity of wording using a four-point scale (1 = not relevant/representative/clear, 4 = highly relevant/representative/clear), with ratings of 3 or 4 considered acceptable. Based on these ratings, three content validity indices were calculated: I-CVI, S-CVI/Ave, and S-CVI/UA (Polit and Beck, 2006). The predefined decision rules were as follows: items with I-CVI = 1.00 were retained; items with I-CVI = 0.83 (5/6 agreement) were retained with revision based on expert feedback; and items with I-CVI ≤ 0.67 were deleted or rewritten. Scales with S-CVI/Ave ≥ 0.90 and S-CVI/UA ≥ 0.80 were considered acceptable (Polit and Beck, 2006). After the first round of expert review, six items that failed to meet the criteria were removed, and several items were revised in accordance with expert suggestions. The remaining 25 items entered a second round of expert review. The results of the second round of review showed that the I-CVI values for relevance, representativeness, and clarity were all 0.83 or 1.00. The corresponding S-CVI/Ave values were 0.97 for all three criteria, and the S-CVI/UA values were 0.84, 0.80, and 0.80, respectively. These results indicate that the scale demonstrates good content validity.

To ensure the validity of the self-developed scale, item analysis was first conducted, and items without significant differences between high and low scoring groups were removed. Subsequently, items with a correlation coefficient between individual item scores and total scores below 0.3 were excluded. To rigorously validate the scale structure and address overfitting concerns, we used convenience sampling to collect data and randomly split the sample into two subsamples. Subsample 1 (*N* = 604) was used for exploratory factor analysis (EFA) to explore and purify the factor structure, whereas Subsample 2 (*N* = 617) was used for confirmatory factor analysis (CFA) to cross-validate the factor structure derived from Subsample 1.

Exploratory factor analysis was then performed on the remaining items. The KMO value was 0.953, and the Bartlett's test of sphericity yielded a chi-square value of 23,754.766, both indicating that the data were suitable for factor analysis. Following factor extraction, items with standardized factor loadings below 0.50 were eliminated. The standardized factor loadings for the retained items ranged from 0.695 to 0.944, demonstrating good structural validity.

Finally, confirmatory factor analysis was conducted (in Subsample 2) for model validation, and items with high covariance were removed. The final structural equation model fit indices were as follows: χ^2^/df = 3.884, RMSEA = 0.076, CFI = 0.952, GFI = 0.907, and TLI = 0.937, indicating an acceptable model fit. The CR for the four dimensions was 0.912, 0.908, 0.892, and 0.932, respectively, and the AVE was 0.724, 0.666, 0.624, and 0.775, respectively, indicating good internal consistency and discriminant validity of the scale (see Table 2). The overall Cronbach's α coefficient for the scale was 0.955, indicating excellent reliability.

**Table 2:** AVE values and inter-factor correlations of latent constructs measuring teachers' STEM literacy.

**Latent variable**	**AVE**	**Development and implementation of STEM curriculum**	**Value orientation toward STEM education**	**Disciplinary foundations and interdisciplinary integration**	**Evaluation of STEM courses**
Development and implementation of STEM curriculum	0.624	**0.790**			
Value orientation toward STEM education	0.724	0.404	**0.851**		
Disciplinary foundations and interdisciplinary integration	0.666	0.603	0.350	**0.816**	
Evaluation of STEM courses	0.775	0.667	0.340	0.524	**0.880**

Bold values on the diagonal indicate the square roots of AVE for each construct; off-diagonal values are inter-construct correlations.

#### 3.2.4 Work engagement scale

The nine-item short version of the *Work Engagement Scale* developed by Schaufeli et al. (2006) was used, covering three dimensions: vigor, dedication, and absorption. Scores can be calculated separately for each dimension or summed for an overall score. Although a formal Chinese version of this scale has not been established, it has been widely applied in domestic research and has demonstrated good reliability and validity. Our preliminary test results indicated weak differentiation among the three dimensions; therefore, we opted to use only the total score without distinguishing between dimensions. After removing items with low factor loadings and high covariance, the final scale comprised four items, presented in a seven-point Likert scale format ranging from “never” to “always” with scores from 0 to 6. Higher scores indicated a higher level of work engagement among teachers. The scale exhibited excellent reliability with a Cronbach's α coefficient of 0.939. Structural equation modeling was employed to assess the scale's validity, yielding a Kaiser-Meyer-Olkin (KMO) measure of sampling adequacy of 0.939 (*p* = 0.000). Standardized factor loadings for the items ranged from 0.847 to 0.931. The structural equation model fit indices were as follows: χ^2^/df = 3.965, RMSEA = 0.049, CFI= 0.999, GFI = 0.997, and TLI = 0.996, indicating a good model fit.

## 4 Results

### 4.1 Descriptive statistics and correlation analysis

Descriptive statistics revealed that teachers' overall level of STEM resistance was relatively low (M = 2.21, SD = 0.72), indicating that most teachers held an open attitude toward STEM education. Regarding the two dimensions of leadership style, teachers perceived a stronger benevolence-moral leadership (M = 3.83, SD = 0.95) compared to authoritarian leadership (M = 2.67, SD = 1.04). This suggests that although a certain degree of authoritarian management exists, the overall leadership style leans toward humanistic care. Teachers' overall STEM literacy was at an above-average level (M = 3.49, SD = 0.70). Teachers also scored high on work engagement (M = 5.57, SD = 1.29), demonstrating strong professional enthusiasm and dedication.

Correlation analysis (see Table 3) revealed that authoritarian leadership showed significant negative correlations with teachers' STEM literacy (*r* = −0.070, *p* < 0.05) and work engagement (*r* = −0.184, *p* < 0.05), and a significant positive correlation with STEM resistance (*r* = 0.347, *p* < 0.001). In contrast, benevolence–moral leadership was positively correlated with STEM literacy (*r* = 0.445, *p* < 0.001) and work engagement (*r* = 0.414, *p* < 0.001), and negatively correlated with STEM resistance (*r* = −0.277, *p* < 0.001). Moreover, teachers' STEM literacy was negatively correlated with their STEM resistance (*r* = −0.411, *p* < 0.001), as was work engagement (*r* = −0.259, *p* < 0.001). These results provide preliminary empirical support for the study's hypotheses.

**Table 3:** Correlation analysis results.

**Variables**	**1**	**2**	**3**	**4**	**5**	**6**	**7**	**8**	**9**	**10**	**11**	**12**
1. Gender												
2. Education background	0.108^***^											
3. Duration of STEM teaching experience	−0.087^**^	−0.142^***^										
4. Professional title	−0.095^**^	−0.208^***^	0.151^***^									
5. Studying phase	−0.215^***^	0.206^***^	0.058^*^	0.006								
6. School location (urban/rural, primary)	−0.142^**^	−0.154^**^	−0.038	0.119^*^	—							
7. School location (urban/rural, junior high)	−0.134^**^	−0.153^**^	0.103^*^	−0.017	—	—						
8. TSL	0.025	0.042	0.092^**^	−0.074^**^	−0.162^***^	−0.089	0.056					
9. WE	0.03	−0.027	0.080^**^	−0.006	−0.061^*^	−0.022	0.059	0.379^***^				
10. AL	−0.181^***^	−0.059^*^	0.107^***^	0.083^**^	0.130^***^	0.079	0.009	−0.070^*^	−0.184^***^			
11. BL	0.130^***^	0.026	−0.044	−0.080^**^	−0.196^***^	−0.099^*^	−0.047	0.445^***^	0.414^***^	−0.400^***^		
12. STR	−0.133^***^	−0.089^**^	0.012	0.114^***^	0.127^***^	0.164^**^	0.046	−0.411^***^	−0.259^***^	0.347^***^	−0.277^***^	
Mean	1.790	2.900	2.300	2.990	1.460	1.390	1.290	3.487	5.570	2.669	3.833	2.209
SD	0.410	0.448	1.843	1.406	0.499	0.489	0.453	0.705	1.292	1.040	0.946	0.725

AL, authoritarian leadership; BL, benevolent–moral leadership; TSL, teacher STEM literacy; WE, work engagement; STR, teacher STEM resistance.

^*^p < 0.05, ^**^p < 0.01, ^***^p < 0.001.

“—” indicates that the correlation is not applicable or cannot be computed.

### 4.2 Hypothesis testing

To comprehensively test the proposed mediated-moderation model in this study, an observed-variable path model was estimated in AMOS using scale scores for authoritarian leadership, benevolence–moral leadership, teachers' STEM literacy, work engagement, and the corresponding leadership × work engagement interaction terms. Model fit and the direct and indirect (mediating) paths were then evaluated. The analysis results indicated that the structural equation model exhibited an excellent fit, with the following fit indices: χ^2^/df = 0.911, RMSEA = 0.000 [90% CI (0.000, 0.055)], CFI = 1.000, GFI = 1.000, and TLI = 1.001. However, considering the limited support of structural equation modeling for confidence interval tests of moderation effects and slope estimations at different levels (e.g., M ± 1SD), this study additionally employed the Hayes' PROCESS macro for SPSS (version 4.2 beta), Model 7, to test moderation effects. This approach utilized the bootstrap method to estimate the significance of moderation pathways and the degree of moderation on indirect effects, thereby enhancing the statistical power to detect interaction effects and effectively compensating for the limitations of structural equation modeling.

Prior to testing the mediating and moderating effects, all variables—including authoritarian leadership, benevolence–moral leadership, teachers' STEM literacy, work engagement, and STEM resistance—were standardized to mitigate multicollinearity. In this model, authoritarian leadership and benevolence-moral leadership served as independent variables, teachers' STEM resistance as the dependent variable, teachers' work engagement as the moderating variable, and teachers' STEM literacy as the mediating variable. Control variables encompassed teachers' gender, educational background, STEM education experience, professional title, and teaching level. Simple slopes were probed at teachers' work engagement values corresponding to the mean and ±1 SD, and inference was based on 5,000 bootstrap resamples with 95% percentile bootstrap confidence intervals.

**Figure 2:**
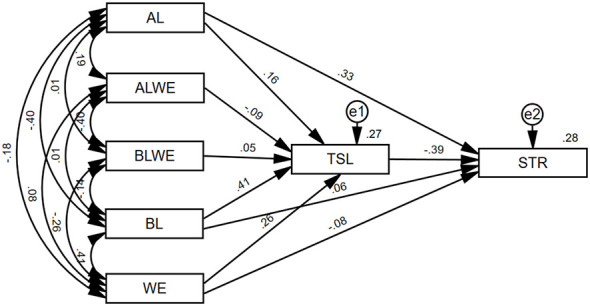
SEM model path.

As shown in Figure 2, the AMOS analysis results indicate that authoritarian leadership is a significant positive predictor of teachers' STEM resistance (β = 0.329, *p* < 0.001), supporting H1. Authoritarian leadership is also a significant positive predictor of teachers' STEM literacy (β = 0.158, *p* < 0.001), which does not support H3. Meanwhile, STEM literacy is a significant negative predictor of teachers' STEM resistance (β =-0.385, *p* < 0.001), supporting H5. The indirect path from authoritarian leadership to teachers' resistance via STEM literacy (0.158 × −0.385 ≈ −0.061) was opposite in direction to the direct path, indicating a suppression pattern in the statistical model, with the direct path coefficient decreasing after the mediator was included. Therefore, teachers' STEM literacy partially mediated the relationship between authoritarian leadership and teachers' STEM resistance, consistent with Hypothesis 6 (H6).

**Figure 3:**
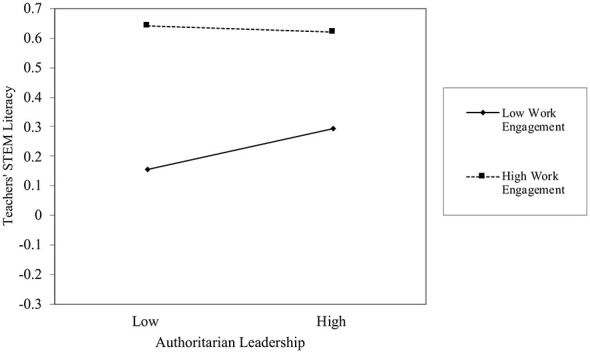
The moderating effect of work engagement on authoritarian leadership and teachers' STEM literacy.

To further examine the mediating role of teachers' STEM literacy in the relationship between authoritarian leadership and teachers' resistance, the PROCESS macro was employed to test the moderating effect. The results revealed a significant interaction between authoritarian leadership and work engagement (β = −0.040, *p* = 0.003), indicating that work engagement significantly moderated the impact of authoritarian leadership on teachers' STEM literacy. Further simple slope analysis (as shown in Table 4) demonstrated that under low levels of work engagement (M – 1SD), authoritarian leadership significantly and positively predicted teachers' STEM literacy; at moderate levels, the effect was not significant; whereas under high levels of work engagement (M + 1SD), the effect reversed. These findings suggest that authoritarian leadership was significantly and positively associated with teachers' STEM literacy only when work engagement was low, whereas a negative association emerged when work engagement was high (as depicted in Figure 3). Thus, work engagement moderated the predictive relationship between authoritarian leadership and teachers' STEM literacy, confirming Hypothesis 9 (H9).

**Table 4:** Results of the moderated mediation effect test for work engagement.

**Independent variable**	**Outcome variable**	**Moderator**	**Level of moderator**	**Indirect effect**	**Standard error**	95% confidence interval	
						**Lower limit**	**Upper limit**
Authoritarian leadership	Teacher's STEM literacy	Work engagement	Low-score group	−0.031	0.012	−0.055	−0.009
			High-score group	0.009	0.010	−0.011	0.029
			Difference between groups	0.040	0.015	0.011	0.070
Benevolence-moral leadership	Teacher's STEM literacy	Work engagement	Low-score group	−0.073	0.012	−0.099	−0.051
			High-score group	−0.105	0.015	−0.136	−0.077
			Difference between groups	−0.033	0.014	−0.061	−0.003

As shown in Figure 2 in the preceding text, benevolence–moral leadership was not a significant predictor of teachers' STEM resistance (β = 0.058, *p* = 0.061), indicating that Hypothesis 2 (H2) was not supported. Benevolence–moral leadership significantly and positively predicted teachers' STEM literacy (β = 0.409, *p* < 0.001), while STEM literacy significantly and negatively predicted teachers' STEM resistance (β = −0.385, *p* < 0.001), confirming Hypotheses 4 (H4) and 5 (H5). Therefore, teachers' STEM literacy fully mediated the relationship between benevolence-moral leadership and teachers' STEM resistance, consistent with Hypothesis 7 (H7). Finally, work engagement significantly and negatively predicted teachers' STEM resistance (β = −0.076, *p* =0.006), supporting Hypothesis 8 (H8).

**Figure 4:**
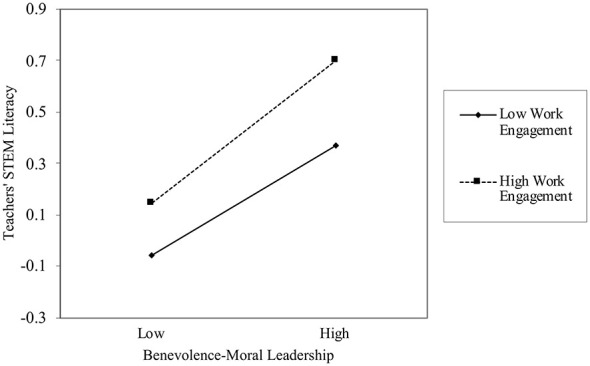
Moderating effect of work engagement on the relationship between benevolence-moral leadership and teachers' STEM literacy.

Further analysis of the moderating effect of work engagement using PROCESS macro Model 7 revealed that the interaction between benevolence-moral leadership and work engagement was significantly associated with teachers' STEM literacy (β = 0.039, *p* = 0.003), indicating that work engagement significantly moderated the relationship between benevolence-moral leadership and teachers' STEM literacy. Further analysis of the moderated mediation effect, as shown in Table 4, demonstrated that as levels of work engagement levels increased, the positive association between benevolence-moral leadership and teachers' STEM literacy became stronger (as depicted in Figure 4), confirming the effective moderating role of work engagement in the relationship between benevolence-moral leadership and teachers' STEM literacy and thereby validating Hypothesis10 (H10).

## 5 Discussion

### 5.1 Paternalistic leadership and teachers' STEM resistance

Paternalistic leadership, rooted in Confucian culture (Farh and Cheng, 2000), combines authority with benevolence (Pang et al., 2012). Some believe that the management art of “combining kindness with strictness” in paternalistic leadership can achieve ideal management outcomes (Farh and Cheng, 2000). This study provides survey-based evidence for the managerial relevance of paternalistic leadership in STEM education, and further shows that authoritarian leadership and benevolence–moral leadership exhibit inconsistent patterns of association. Authoritarian leadership significantly and positively predicted teachers' STEM resistance, whereas benevolence-moral leadership did not significantly negatively predict it. This finding suggests that paternalistic leadership is not consistently associated with positive outcomes in STEM education reform. Specifically, authoritarian leadership, which emphasizes hierarchy and obedience, is positively associated with teacher resistance and higher levels of opposition to STEM teaching changes. Although benevolent–moral leadership is characterized by emotional support and care and is negatively associated with teacher resistance, its direct association is not statistically significant. Previous research findings align with this study: for example, Long et al. (2014) found that authoritarian leadership directly and positively predicted work alienation, while benevolent leadership did not significantly and negatively predict it directly but exerted an influence through organizational support as a mediator. Similarly, research on university teachers has revealed that job satisfaction fully mediated the relationship between moral leadership and organizational citizenship behavior, as well as the relationship between benevolent leadership and task performance (Qiu and Yang, 2015). Thus, the functioning of paternalistic leadership in management appears to be complex, with different leadership styles being related with teachers' attitudes and behaviors through distinct pathways. The positive associations of benevolence–moral leadership may be latent, requiring mediation through other psychological processes before becoming observable in empirical models.

### 5.2 Teachers' STEM literacy as a mediator

First, STEM literacy fully mediated the relationship between benevolence-moral leadership and teachers' STEM resistance. This study found that although the direct association between benevolence–moral leadership and teacher resistance was nonsignificant, a significant indirect path was observed via teachers' STEM literacy. Specifically, benevolence–moral leadership was positively predicted with teachers' STEM literacy, which in turn was negatively associated with teacher resistance. This result reveals that benevolence-moral leadership is not directly related to lower resistance to change but is indirectly associated with lower resistance through its association with teachers' professional competence, which is consistent with the “resource gain” logic of Conservation of Resources Theory (Hobfoll et al., 2018). That is, benevolence-moral leadership creates psychological security and a positive work attitude for teachers through caring support and moral exemplarity (Zheng et al., 2020), prompting them to translate leadership support into motivation for improving STEM literacy and thereby alleviating resistance to change due to insufficient competence. This finding expands current understanding of the pathways through which benevolence-moral leadership is associated with educational outcomes, suggesting that its role is more strongly reflected in capacity building rather than in the direct transmission of pressure.

Second, STEM literacy partially mediated the relationship between authoritarian leadership and teachers' STEM resistance. This study found that authoritarian leadership significantly and positively predicted teachers' STEM resistance, indicating that authoritative and command-oriented management is associated with higher levels of teacher resistance and dissatisfaction. On the other hand, although authoritarian leadership significantly and positively predicted teachers' STEM literacy, the pathway analysis revealed characteristics of a “suppression effect” (MacKinnon et al., 2000). Specifically, authoritarian leadership was indirectly associated with lower teacher resistance through its positive association with STEM literacy; however, this indirect pathway was opposite in direction to its positive direct path, resulting in a partial mediating role overall. This finding suggests that although authoritarian leadership is related to higher levels of teachers' professional performance indicators (e.g., skills training and teaching standardization embedded in institutional arrangements), its relatively limited psychological support and emotional connection are also associated with higher levels of teacher resistance. This contradictory effect resonates with leadership research on the “paradoxical behavior”^1^ (Batool et al., 2023), underscoring the need for caution: short-term improvements in teachers' STEM literacy under authoritarian leadership may be accompanied by long-term psychological depletion.

### 5.3 Work engagement as a predictor and moderator

This study found that work engagement significantly and negatively predicted teachers' STEM resistance, meaning that higher work engagement was associated with less resistance and fewer resistant behaviors toward STEM teaching changes among teachers. Teachers with high work engagement tend to be more focused, enthusiastic, and responsible, and are more likely to report supportive attitudes toward teaching reforms; correspondingly, higher work engagement is associated with lower levels of resistance to new teaching concepts and methods. Although work engagement is often viewed as an outcome variable (Christian et al., 2011; Kim et al., 2013), research shows that it forms a gain cycle with factors such as willingness to change, with mutual causation (van den Heuvel et al., 2010). According to Conservation of Resources Theory (Hobfoll et al., 2018), teachers with high work engagement tend to actively invest their personal resources (e.g., psychological capital, professional efficacy) into their work to enhance their adaptability and cope with challenges brought by change. This study confirms from an educational perspective that work engagement is an antecedent variable of teacher resistance, influencing their attitudes toward teaching changes.

This study also found that work engagement played a differentiated moderating role under different leadership styles. The positive association between benevolence–moral leadership and STEM literacy became stronger as work engagement increased, with the moderating effect of work engagement exhibiting a relatively stable gain effect. In other words, the positive association between benevolence–moral leadership and teachers' STEM literacy was stronger at higher levels of work engagement. Previous research has found that when teachers perceive higher supervisor support, the positive correlation between work engagement and their perceived work competence is stronger; conversely, this correlation is weaker when supervisor support is perceived as low (Lin et al., 2022). This study further confirmed this relationship from the perspective of STEM education, clarifying the pattern of associations among teachers' work engagement, benevolence–moral leadership, and teachers' STEM literacy. At the same time, it is consistent with the motivational pathway of the Job Demands–Resources (JD–R) model (Bakker and Demerouti, 2007): benevolence–moral leadership is associated with higher levels of perceived job resources, such as emotional support, moral guidance, and organizational support, which in turn are related to higher work engagement. Teachers with higher levels of engagement also tend to report more learning-oriented behaviors and higher levels of STEM literacy, suggesting a mutually reinforcing pattern among leadership support, engagement, and professional development.

Conversely, authoritarian management was positively associated with STEM literacy only when teachers' work engagement was low, whereas this association became weaker or even negative as work engagement increased. When work engagement was low, authoritarian leadership was positively and significantly associated with teachers' STEM literacy; however, when work engagement was moderate or high, this positive association gradually weakened and even became negative, displaying an “inverted U-shaped” moderating trend. A possible reason is that when teachers have low work engagement, authoritarian leadership can lead to limited improvements in competence through pressure-driven compliance with professional development management; however, for highly engaged teachers, authoritarian management may suppress their autonomy and creativity, thereby weakening their motivation for professional development. Previous research has also confirmed from aspects such as employee compliance, gratitude, and supervision satisfaction that the impact of leaders' authoritarianism on subordinates varies depending on employees' “traditionality”^2^ or authority orientation: for subordinates with high authority orientation and who value compliance and obligations, authoritarian leadership shows a larger effect. For subordinates who stress equal rights, the association tends to weaken or become non-significant among subordinates with low authority orientation (Cheng et al., 2004). Self-Determination Theory suggests that when external control exceeds an individual's capacity for autonomous regulation, even if the target outcome (e.g., STEM literacy) temporarily improves, it may still lead to motivation depletion and reverse resistance due to violation of the individual's fundamental need for autonomy (Deci and Ryan, 2000). This complex effect is particularly salient in the field of STEM education: in many countries, including China, there are no specialized pre-service teacher education programs for STEM teachers. Consequently, closing institutional gaps largely depends on the continuous learning and professional initiative of in-service teachers. If leaders adopt high-pressure authoritarian management to promote reforms, it may produce only superficial or compliant changes in the short term, but fail to foster teachers' deep identification with and sustained commitment to STEM education. Instead, such practices risk suppressing teachers' intrinsic motivation for professional growth and undermining the long-term effectiveness of reforms.

### 5.4 Limitations and further work

Although this study has made certain explorations in theoretical construction and empirical analysis, it still has limitations that need to be further addressed in future research:

First, the research design mainly relies on cross-sectional data, making it difficult to reveal causal mechanisms among variables. This study uncovered the pathways of influence among leadership styles, teachers' STEM literacy, work engagement, and teachers' STEM resistance through structural equation modeling and mediation-moderation analysis. However, since the data were collected at a single time point, it is challenging to confirm the temporal sequence and dynamic changes among the variables. Future research could consider adopting longitudinal designs or experimental studies to more precisely identify causal relationships and developmental trends.

Second, although the research results show a significant moderating effect of work engagement, the underlying mechanisms remain to be further explored. This study found that work engagement exerts either amplifying or suppressing effects on the pathways through which different leadership styles influence outcomes; however, the underlying mechanisms driving these effects remain unclear. In particular, school organizational contexts may also influence leadership effectiveness beyond the factors involved in this study. For example, a school's innovative culture may strengthen the transmission effect of benevolence-moral leadership on STEM-related resources, while administrative accountability or standardized testing pressures may amplify the negative effects of authoritarian leadership. Therefore, it is still necessary to further integrate organizational-level antecedent variables and individual-level psychological processes to analyze their mechanisms of action.

Third, the sample source has certain regional limitations, which may affect the generalizability of the research conclusions. The sample for this study was selected from STEM teachers in a provincial capital city in northern China. Due to the influence of cultural, institutional, and economic development levels, the research conclusions have certain situational dependencies. Future research could expand the sample coverage and conduct cross-regional or even cross-cultural comparative studies to enhance the universal applicability and cross-situational explanatory power of the research conclusions.

## 6 Conclusions

This study examined the relationships among paternalistic leadership, STEM literacy, work engagement, and teachers' STEM resistance. The findings indicate that authoritarian leadership was positively associated with teachers' resistance, whereas benevolence–moral leadership was indirectly associated with lower resistance via STEM literacy. These patterns suggest that different leadership styles may operate through distinct psychological and professional processes, which may have important implications for managing teacher resistance during STEM reform. STEM literacy emerged as a central mediator: it fully transmitted the effect of benevolence-moral leadership and partially mediated the paradoxical effect of authoritarian leadership. Although authoritarian leadership predicted higher STEM literacy, path analysis showed a suppression effect: the indirect pathway—in which higher STEM literacy was associated with lower resistance—ran opposite to the direct pathway, in which authoritarian leadership was associated with higher resistance. This pattern reflects a suppression effect with partial mediation; short-term improvements in teachers' STEM literacy observed in association with authoritarian leadership may be accompanied by long-term psychological depletion. Moreover, work engagement not only negatively predicted resistance but also moderated leadership effects. For benevolence–moral leadership, the positive association with teachers' STEM literacy was stronger at higher levels of work engagement, while the effect of authoritarian leadership followed an inverted U-shaped trend, turning from positive under low engagement to negative at higher engagement. To enhance teachers' STEM literacy and willingness to participate in STEM education, schools may consider cultivating a culture of benevolent-moral leadership while carefully defining the boundaries of authoritarian management practices. Meanwhile, differentiated incentive and support mechanisms should be developed based on teachers' levels of work engagement, thereby fostering sustained professional development and active involvement in STEM education reform, which provides important implications for the management of STEM reform at the school level.

## Data Availability

The raw data supporting the conclusions of this article will be made available by the authors, without undue reservation.

## Footnotes

1 A leader's apparently competing yet interrelated behaviors to meet structural and follower demands is known as paradoxical behavior.

2 Here, “traditionality” refers to the tendency of employees to accept and follow the hierarchical system in society, such as whether they tend to obey authority and follow the superior-subordinate relationship.

## References

[B1] Achinstein B. Ogawa R. T. (2006). In(fidelity): What the resistance of new teachers reveals about professional principles and prescriptive educational policies. Harv. Educ. Rev. 76, 30–63. doi: 10.17763/haer.76.1.e14543458r811864

[B2] Al Salami M. K. Makela C. J. de Miranda M. A. (2017). Assessing changes in teachers' attitudes toward interdisciplinary STEM teaching. Int. J. Technol. Des. Educ. 27, 63–88. doi: 10.1007/s10798-015-9341-0

[B3] Alanoglu M. Aslan S. Karabatak S. (2022). Do teachers' educational philosophies affect their digital literacy? The mediating effect of resistance to change. Educ. Inf. Technol. 27, 3447–3466. doi: 10.1007/s10639-021-10753-3 34602847 PMC8475411

[B4] Armenakis A. A. Harris S. G. Mossholder K. W. (1993). Creating readiness for organizational change. Hum. Relat. 46, 681–703. doi: 10.1177/001872679304600601

[B5] Aycan Z. (2006). “Paternalism: Towards conceptual refinement and operationalization,” in Indigenous and Cultural Psychology: Understanding People in Context, ed. U. Kim, K. S. Yang, and K. K. Hwang (New York, NY: Springer), 445–466.

[B6] Bakker A. B. Demerouti E. (2007). The job demands-resources model: state of the art. J. Manag. Psychol. 22, 309–328. doi: 10.1108/02683940710733115

[B7] Balgopal M. M. (2020). STEM teacher agency: a case study of initiating and implementing curricular reform. Sci. Educ. 104, 762–785. doi: 10.1002/sce.21578

[B8] Ballet K. Kelchtermans G. (2009). Struggling with workload: primary teachers' experience of intensification. Teach. Teach. Educ. 25, 1150–1157. doi: 10.1016/j.tate.2009.02.012

[B9] Bass B. M. Riggio R. E. (2006). Transformational Leadership, 2nd Edn. New York, NY: Psychology Press.

[B10] Batool U. Raziq M. M. Sarwar N. (2023). The paradox of paradoxical leadership: a multi-level conceptualization. Hum. Resour. Manage. Rev. 33:100983. doi: 10.1016/j.hrmr.2023.100983

[B11] Bedi A. (2020). A meta-analytic review of paternalistic leadership. J. Organ. Behav. 41, 960–1008. doi: 10.1111/apps.12186

[B12] Benett Y. (1980). Teachers' attitudes to curriculum innovation: making explicit a psychological perspective. Vocat. Asp. Educ. 32, 71–76. doi: 10.1080/10408347308001381

[B13] Biesta G. Priestley M. Robinson S. (2017). Talking about education: exploring the significance of teachers' talk for teacher agency. J. Curric. Stud. 49, 38–54. doi: 10.1080/00220272.2016.1205143

[B14] Breevaart K. Bakker A. Hetland J. Demerouti E. Olsen O. K. Espevik R. (2014). Daily transactional and transformational leadership and daily employee engagement. J. Occup. Organ. Psychol. 87, 138–157. doi: 10.1111/joop.12041

[B15] Bridwell-Mitchell E. N. Sherer D. G. (2017). Institutional complexity and policy implementation: how underlying logics drive teacher interpretations of reform. Educ. Eval. Policy Anal. 39, 223–247. doi: 10.3102/0162373716677567

[B16] Cai Y. Qu Y. Tang R. (2024). 家长式领导行为与教师专业学习共同体的关系: 人际信任的作用 [The relationship between paternalistic leadership behaviors and teachers' professional learning communities: the role of interpersonal trust]. 教育科学研究 [Educ. Sci. Res.] 3, 76–83. Chinese.

[B17] Caniëls M. C. J. Semeijn J. H. Renders I. H. M. (2018). Mind the mindset! The interaction of proactive personality, transformational leadership and growth mindset for engagement at work. Career Dev. Int. 23, 48–66. doi: 10.1108/CDI-11-2016-0194

[B18] Chan S. C. H. Huang X. Snape E. D. Lam C. K. (2013). The Janus face of paternalistic leaders: authoritarianism, benevolence, subordinates' organization-based self-esteem, and performance. J. Organ. Behav. 34, 108–128. doi: 10.1002/job.1797

[B19] Chang I.-H. (2012). The effect of principals' technological leadership on teachers' technological literacy and teaching effectiveness in Taiwanese elementary schools. Educ. Technol. Soc. 15, 328–340.

[B20] Chen L. Yang B. Jing R. (2015). Paternalistic leadership, team conflict, and TMT decision effectiveness: interactions in the Chinese context. Manage. Organ. Rev. 11, 739–762. doi: 10.1017/mor.2015.34

[B21] Cheng B.-S. Chou L.-F. Wu T.-Y. Huang M.-P. Farh J.-L. (2004). Paternalistic leadership and subordinate responses: establishing a leadership model in Chinese organizations. Asian J. Soc. Psychol. 7, 89–117. doi: 10.1111/j.1467-839X.2004.00137.x

[B22] Cheng B. S. (1995). 家长权威与领导行为之关系:一个台湾民营企业主持人的个案研究 [Paternalistic authority and leadership: a case study of a Taiwanese CEO]. 中央研究院民族学研究所 *[Bull. Inst. Ethnol. Acad. Sin*.] 79, 119–173. Chinese.

[B23] Cheng B. S. Chou L. F. Farh J. L. (2000). 家长式领导:三元模式的建构与测量 [A triad model of paternalistic leadership: constructs and measurement]. 本土心理学研究 *[Indig. Psychol. Res. Chin. Soc*.] 14, 3–64. Chinese. doi: 10.6254/IPRCS.200012_(14).0001

[B24] Christian M. S. Garza A. S. Slaughter J. E. (2011). Work engagement: a quantitative review and test of its relations with task and contextual performance. Pers. Psychol. 64, 89–136. doi: 10.1111/j.1744-6570.2010.01203.x

[B25] Coch L. French J. R. P. (1948). Overcoming resistance to change. Hum. Relat. 1, 512–532. doi: 10.1177/001872674800100408

[B26] Deci E. L. Ryan R. M. (2000). The “what” and “why” of goal pursuits: human needs and the self-determination of behavior. Psychol. Inq. 11, 227–268. doi: 10.1207/S15327965PLI1104_01

[B27] Deluma R. Zulela M. S. Asmawi M. (2020). The effect of principal leadership style, work motivation, and professional competence of primary school teacher performance in Kendari City. J. Educ. Teach. Learn. 5, 145–151. doi: 10.26737/jetl.v5i1.1134

[B28] Desianti L. C. Hardhienata S. Setyaningsih S. (2023). The modelling of ICT literacy, work engagement, and personal knowledge management to enhance teacher creativity. Asian J. Manage. Entrep. Soc. Sci. 3, 164–192. doi: 10.63922/ajmesc.v3i03.349

[B29] Dong Z. (2016). 试论我国中小学实施STEM课程的困境与对策 [On the dilemmas and countermeasures of implementing STEM curricula in Chinese primary and secondary schools]. 全球教育展望 [Glob. Educ. Outlook] 5, 36–42, 62. Chinese.

[B30] Edmondson A. (1999). Psychological safety and learning behavior in work teams. Adm. Sci. Q. 44, 350–383. doi: 10.2307/2666999

[B31] Escudeiro P. Escudeiro N. Campos M. (2024). Empowering education: the STEAME project's vision for teacher competence and innovation in STEM learning environments. INTED2024 Proc. 3256–3261. doi: 10.21125/inted.2024.0870

[B32] Farh J.-L. Cheng B.-S. (2000). “A cultural analysis of paternalistic leadership in Chinese organizations,” in Management and Organizations in the Chinese Context, ed. J. T. Li, A. S. Tsui and E. Weldon (London: Palgrave Macmillan), 84–127.

[B33] Fullan M. (2007). The New Meaning of Educational Change, 4th Edn. New York, NY: Teachers College Press.

[B34] Fullan M. Pomfret A. (1977). Research on curriculum and instruction implementation. Rev. Educ. Res. 47, 335–397. doi: 10.3102/00346543047002335

[B35] Galleon J. M. F. De Leon M. G. M. (2023). The influence of academic competence and well-being on work engagement of elementary school teachers. Int. J. Res. Publ. Rev. 4, 2209–2216.

[B36] Geijsel F. P. Sleegers P. J. C. Leithwood K. Jantzi D. (2003). Transformational leadership effects on teachers' commitment and effort toward school reform. J. Educ. Adm. 41, 228–256. doi: 10.1108/09578230310474403

[B37] Gorgievski M. Hobfoll S. E. (2008). “Work can burn us out and fire us up,” in Handbook of Stress and Burnout in Health Care, ed. J. R. B. Halbesleben (New York, NY: Nova Science Publishers), 7–22.

[B38] Guskey T. R. (2002). Professional development and teacher change. Teachers and Teaching 8, 381–391. doi: 10.1080/135406002100000512

[B39] Hall D. McGinity R. (2015). Conceptualizing teacher professional identity in neoliberal times: resistance, compliance and reform. Educ. Policy Anal. Arch. 23:88. doi: 10.14507/epaa.v23.2092

[B40] Hall G. E. Hord S. M. (2015). Implementing Change: Patterns, Principles, and Potholes, 4th Edn. Boston, MA: Pearson.

[B41] Hall G. E. Wu X. (2004). 实施变革*:* 模式原则与困境 *[Implementing change: patterns, principles, and potholes]*. Hangzhou: Zhejiang Education Press. Chinese.

[B42] Han D. (2011). 教师阻抗学校变革的理性思考 [Rational reflections on teachers' resistance to school change]. 当代教育科学 [Contemp. Educ. Sci.] 1, 3–6. Chinese.

[B43] Hargreaves A. (2005). Educational change takes ages: life, career, and generational factors in teachers' emotional responses to educational change. Teach. Teach. Educ. 21, 967–983. doi: 10.1016/j.tate.2005.06.007

[B44] Hasanah U. (2020). Key definitions of STEM education: literature review. Interdiscip. J. Environ. Sci. Educ. 16:e2217. doi: 10.29333/ijese/8336

[B45] Hasti A. Purwanto N. A. (2024). The influence of principal leadership, organizational culture and interpersonal communication on teacher competence. Int. J. Educ. Technol. Res. 2, 756–767. doi: 10.59890/ijetr.v2i4.284

[B46] Hiller N. J. Sin H.-P. Ponnapalli A. R. Özgen S. (2019). Benevolence and authority as WEIRDly unfamiliar: a multi-language meta-analysis of paternalistic leadership behaviors from 152 studies. Leadh. Q. 30, 165–184. doi: 10.1016/j.leaqua.2018.11.003

[B47] Hobfoll S. E. (1989). Conservation of resources: a new attempt at conceptualizing stress. Am. Psychol. 44, 513–524. doi: 10.1037/0003-066X.44.3.513 2648906

[B48] Hobfoll S. E. Halbesleben J. Neveu J.-P. Westman M. (2018). Conservation of resources in the organizational context: the reality of resources and their consequences. Annu. Rev. Organ. Psychol. Organ. Behav. 5, 103–128. doi: 10.1146/annurev-orgpsych-032117-104640

[B49] Hofstede G. (2001). Culture's Consequences: Comparing Values, Behaviors, Institutions, and Organizations Across Nations, 2nd Edn. Thousand Oaks, CA: Sage Publications.

[B50] Hontake T. Ariyoshi H. (2016). A study on work engagement among nurses in Japan: the relationship to job-demands, job-resources, and nursing competence. J. Nurs. Educ. Pract. 6, 111–117. doi: 10.5430/jnep.v6n5p111

[B51] Howard S. K. (2013). Risk-aversion: understanding teachers' resistance to technology integration. Technol. Pedagog. Educ. 22, 357–372. doi: 10.1080/1475939X.2013.802995

[B52] Huang Y.-S. Asghar A. (2018). Science education reform in Confucian learning cultures: teachers' perspectives on policy and practice in Taiwan. Cult. Stud. Sci. Educ. 13, 101–131. doi: 10.1007/s11422-016-9762-4

[B53] Huddleston A. P. Talley S. Edgington S. Colwell E. Dale A. (2024). Teachers' principled resistance to curricular control: a theoretical literature review. Rev. Educ. Res. 95, 1213–1250. doi: 10.3102/00346543241291835

[B54] Iba Z. Mukhtar M. Kamaruddin K. (2023). The influence of principal leadership, competence, and work motivation of teacher on teacher performance at SMA Negeri 1 Tanah Jambo Ayee, District Aceh Utara. Manage. Educ. J. Manaj. Pendidik. Islam 9, 323–336. doi: 10.18592/moe.v9i2.10936

[B55] Isah S. Ibrahim R. M. Shehu M. Uzairu M. G. (2021). The mediating effect of work engagement in the link between affective commitment and employee competence in Nigeria Universal Basic Education System. *Int. Sch. J. Arts Soc. Sci*. Res. 4, 285–305.

[B56] Jeong S. Hsiao Y.-Y. Song J. H. Kim J. Bae S. H. (2016). The moderating role of transformational leadership on work engagement: the influences of professionalism and openness to change. Hum. Resour. Dev. Q. 27, 489–516. doi: 10.1002/hrdq.21265

[B57] Johnson M. J. Jiang L. (2017). Reaping the benefits of meaningful work: the mediating versus moderating role of work engagement. Stress Health 33, 288–297. doi: 10.1002/smi.2710 27647548

[B58] Kahn W. A. (1992). To be fully there: psychological presence at work. Hum. Relat. 45, 321–349. doi: 10.1177/001872679204500402

[B59] Kelchtermans G. (2005). Teachers' emotions in educational reforms: self-understanding, vulnerable commitment and micropolitical literacy. Teach. Teach. Educ. 21, 995–1006. doi: 10.1016/j.tate.2005.06.009

[B60] Kim W. Kolb J. A. Kim T. (2013). The relationship between work engagement and performance: a review of empirical literature and a proposed research agenda. Hum. Resour. Dev. Rev. 12, 248–276. doi: 10.1177/1534484312461635

[B61] Latif Z. Riaz A. Ajmi M. A. Nadeem M. A. Srinivas K. Hasan M. K. (2024). Unraveling the paradox: facades of conformity amid servant leadership and employee readiness to change. Emp. Responsibilities Rights J. doi: 10.1007/s10672-024-09516-2

[B62] Leithwood K. Sun J. (2012). The nature and effects of transformational school leadership: a meta-analytic review of unpublished research. Educ. Adm. Q. 48, 387–423. doi: 10.1177/0013161X11436268

[B63] Lin S.-H. Lu W.-C. Chen Y.-C. Wu M.-H. (2022). The relationships among proactive personality, work engagement, and perceived work competence in sports coaches: the moderating role of perceived supervisor support. Int. J. Environ. Res. Public Health 19:12707. doi: 10.3390/ijerph191912707 36232008 PMC9564478

[B64] Liu Y. (2008). 教师在课程改革中的抗拒 [Teachers' resistance in curriculum reform]. 教育学报 [J. Educ.] 1, 32–36. Chinese. doi: 10.14082/j.cnki.1673-1298.2008.01.009

[B65] Long L. Zhang Y. Liu J. (2014). 家长式领导对员工工作疏离感的影响:组织支持感的中介作用 [The effect of paternalistic leadership on employees' work alienation: the mediating role of perceived organizational support]. [Chin. J. Manage.] 11, 1150–1157. Chinese.

[B66] Lu J. Wen Y. (2005). “新课改”中教师阻抗的文化检视 [A cultural examination of teachers' resistance in new curriculum reform]. 江西教育科研 [Jiangxi Educ. Res.] 10, 45–47. Chinese. doi: 10.16477/j.cnki.issn1674-2311.2005.10.023

[B67] MacKinnon D. P. Krull J. L. Lockwood C. M. (2000). Equivalence of the mediation, confounding and suppression effect. Prev. Sci. 1, 173–181. doi: 10.1023/A:1026595011371 11523746 PMC2819361

[B68] Margot K. C. Kettler T. (2019). Teachers' perception of STEM integration and education: a systematic literature review. Int. J. STEM Educ. 6, 1–16. doi: 10.1186/s40594-018-0151-2

[B69] Masry-Herzalah A. Dor-Haim P. (2022). Teachers' technological competence and success in online teaching during the COVID-19 crisis: the moderating role of resistance to change. Int. J. Educ. Manage. 36, 1–13. doi: 10.1108/IJEM-03-2021-0086

[B70] Matthysen M. Harris C. (2018). The relationship between readiness to change and work engagement: a case study in an accounting firm undergoing change. SA J. Hum. Resour. Manage. 16, 1–11. doi: 10.4102/sajhrm.v16i0.855

[B71] McLaughlin M. W. (1976). Implementation as mutual adaptation: change in classroom organization. Teach. Coll. Rec. 77, 339–351. doi: 10.1177/016146817607700304

[B72] National Institute of Education Sciences Center for STEM Education (2018). STEM教师能力等级标准（试行） *[STEM teacher competency level standards (trial)]*. Beijing: National Institute of Education Sciences Center for STEM Education. Chinese. Available online at: https://stem.zjnu.edu.cn/_upload/article/files/36/b6/14e480444ba6aa3ce68c076d8fcd/0595fe27-c959-4e31-af28-c06e9f6fc07c.pdf (accessed January 14, 2026).

[B73] National Research Council (2012). A Framework for K-12 Science Education: Practices, Crosscutting Concepts, and Core Ideas. Washington, DC: The National Academies Press.

[B74] Oliver A. I. (1977). Curriculum Improvement: A Guide to Problems, Principles, and Process. New York, NY: Joanna Cotler Books.

[B75] Oreg S. (2006). Personality, context, and resistance to organizational change. Eur. J. Work Organ. Psychol. 15, 73–101. doi: 10.1080/13594320500451247

[B76] Oreg S. (2018). Resistance to change and performance: toward a more even-handed view of dispositional resistance. J. Appl. Behav. Sci. 54, 88–107. doi: 10.1177/0021886317741867

[B77] Pang X. Zou G. Song F. (2012). 家长式领导风格与高管团队行为整合的关系 [The relationship between paternalistic leadership styles and top management team behavioral integration]. 中国流通经济 [China Bus. Mark.] 26, 110–114. Chinese. doi: 10.14089/j.cnki.cn11-3664/f.2012.05.021

[B78] Parra-Perez L. G. Gloeckner G. W. Valdés-Cuervo A. A. Addo R. Harindranathan P. (2022). Mexican education reform: elucidating dissenting teachers' resistance. Discourse 43, 115–129. doi: 10.1080/01596306.2020.1811956

[B79] Pei M. Yang H. (2019). Developing teacher agency and self-regulation in a professional training program: a case study in a rural and ethnic minority area of China. Asia Pac. Educ. Rev. 20, 625–639. doi: 10.1007/s12564-019-09606-z

[B80] Pellegrini E. K. Scandura T. A. (2008). Paternalistic leadership: a review and agenda for future research. J. Manage. 34, 566–593. doi: 10.1177/0149206308316063

[B81] Piderit S. K. (2000). Rethinking resistance and recognizing ambivalence: a multidimensional view of attitudes toward an organizational change. Acad. Manage. Rev. 25, 783–794. doi: 10.2307/259206

[B82] Polit D. F. Beck C. T. (2006). The content validity index: are you sure you know what's being reported? Critique and recommendations. Res. Nurs. Health 29, 489–497. doi: 10.1002/nur.20147 16977646

[B83] Poole W. (1991). Resistance to change in education: themes in the literature. Graduate student paper. Syracuse University, Syracuse, NY, United States. ERIC Document No. *ED*330307.

[B84] Priestley M. Biesta G. Robinson S. (2015). Teacher Agency: An Ecological Approach. New York, NY: Bloomsbury.

[B85] Qiu Y. Yang X. (2015). 家长式领导对高校教师工作行为的影响研究–基于任务绩效和组织公民行为的差异视角 [The impact of paternalistic leadership on university teachers' work behavior: a differential perspective of task performance and organizational citizenship behavior]. 复旦教育论坛 [Fudan Educ. Forum] 13, 62–71. Chinese. doi: 10.13397/j.cnki.fef.2015.06.011

[B86] Rahmadani R. Rahim A. R. Prasilowati S. L. Siradjuddin S. (2024). A literature review on the effect of perceived organizational support on employee engagement and employee performance of government agencies in Singapore, Thailand and Indonesia. Int. J. Soc. Serv. Res. 4, 79–96. doi: 10.46799/ijssr.v4i01.663

[B87] Rodriguez A. J. (1998). Strategies for counterresistance: toward sociotransformative constructivism and learning to teach science for diversity and for understanding. J. Res. Sci. Teach. 35, 589–622. doi: 10.1002/(SICI)1098-2736(199808)35:6<589::AID-TEA2>3.0.CO;2-I

[B88] Rong Z. (2007). 抵制, 规避还是适应胜任?—论新基础教育课程改革实施中的教师问题 [Resistance, avoidance, or adaptation and competence? On teacher issues in the implementation of new basic education curriculum reform]. 江西教育科研 [Jiangxi Educ. Res.] 3, 105–107. Chinese. doi: 10.16477/j.cnki.issn1674-2311.2007.03.038

[B89] Sang G. Wang K. Li S. Xi J. Yang D. (2023). Effort expectancy mediates the relationship between instructors' digital competence and their work engagement: evidence from universities in China. Educ. Technol. Res. Dev. 71, 99–115. doi: 10.1007/s11423-023-10205-4 36785812 PMC9907197

[B90] Sannino A. (2010). Teachers' talk of experiencing: conflict, resistance and agency. Teach. Teach. Educ. 26, 838–844. doi: 10.1016/j.tate.2009.10.021

[B91] Schaufeli W. B. Bakker A. B. Salanova M. (2006). The measurement of work engagement with a short questionnaire: a cross-national study. Educ. Psychol. Meas. 66, 701–716. doi: 10.1177/0013164405282471

[B92] Schaufeli W. B. Salanova M. González-Romá V. Bakker A. B. (2002). The measurement of engagement and burnout: a two-sample confirmatory factor analytic approach. J. Happiness Stud. 3, 71–92. doi: 10.1023/A:1015630930326

[B93] Sera-Sirven J. (2021). Veteran teachers' resistance to integrating new technology: a case study. (doctoral dissertation). Northcentral University, San Diego, CA.

[B94] Sheth M. Pathak R. (2023). “STEM education: an interdisciplinary and integrated approach of teaching,” in Interdisciplinary Approaches and Strategies for Sustainable Development, ed. *E. Mundhe* (Geneva: Zenodo).

[B95] Shieh W. (1996). Environmental factors, principal's change facilitator style and implementation of the cooperative learning project in selected schools in Taiwan. (doctoral dissertation). University of Northern Colorado, Greeley, CO.

[B96] Siekmann G. (2016). What is STEM? The need for unpacking its definitions and applications. National Centre for Vocational Education Research. Available online at: https://www.ncver.edu.au/research-and-statistics/publications/all-publications/what-is-stem-the-need-for-unpacking-its-definitions-and-applications

[B97] Song F. Li Y. Zhang Q. (2005). 新课程实施中教师阻抗因素的调查及对策研究 [A survey on teachers' resistance factors in new curriculum implementation and corresponding countermeasures]. 教育探索 [Educ. Explor.] 7, 21–24. Chinese.

[B98] Song F. Su L. (2004). 高校网络教学中教师阻抗因素探析 [An analysis of teachers' resistance factors in online teaching in higher education]. 江苏高教 [Jiangsu High. Educ.] 4, 58–60. Chinese. doi: 10.13236/j.cnki.jshe.2004.04.020

[B99] Song G. Guan J. (2022). 面向整合式STEM的教师跨学科素养: 结构模型与发展路径 [Teachers' interdisciplinary literacy for integrated STEM: a structural model and development path]. 现代远程教育研究 [Mod. Distance Educ. Res.] 34, 58–66. Chinese.

[B100] Spector B. Burkett R. S. Leard C. (2007). Mitigating resistance to teaching science through inquiry: studying self. J. Sci. Teach. Educ. 18, 185–208. doi: 10.1007/s10972-006-9035-2

[B101] Susanti N. Faizah H. Sinaga M. Ijayani I. (2023). The influence of principal transformational leadership and teacher digital literacy on state high school teacher professionalism. Al-Ishlah: J. Pendidik. 15, 246–258. doi: 10.51574/ijrer.v5i1.4258

[B102] Terhart E. (2013). Teacher resistance against school reform: reflecting an inconvenient truth. School Leadh. Manage. 33:486–500. doi: 10.1080/13632434.2013.793494

[B103] Tims M. Bakker A. B. Xanthopoulou D. (2011). Do transformational leaders enhance their followers' daily work engagement? Leadh. Q. 22, 121–131. doi: 10.1016/j.leaqua.2010.12.011

[B104] van den Heuvel M. Demerouti E. Bakker A. B. Schaufeli W. B. (2010). “Personal resources and work engagement in the face of change,” in Contemporary Occupational Health Psychology: Global Perspectives on Research and Practice, Vol. 1, eds. J. Houdmont and S. Leka (Chichester: Wiley-Blackwell), 124–150. doi: 10.1002/9780470661550.ch7

[B105] van Dierendonck D. (2011). Servant leadership: a review and synthesis. J. Manage. 37, 1228–1261. doi: 10.1177/0149206310380462

[B106] Wan M. Wang P. (2005). 教学改革中的文化冲击与文化适应问题 [Cultural shock and adaptation in teaching reform]. 教育研究 [Educ. Res.] 10, 44–48. Chinese.

[B107] Wang A.-C. Tsai C.-Y. Dionne S. D. Yammarino F. J. Spain S. M. Ling H.-C. . (2018). Benevolence-dominant, authoritarianism-dominant, and classical paternalistic leadership: testing their relationships with subordinate performance. Leadh. Q. 29, 686–697. doi: 10.1016/j.leaqua.2018.06.002

[B108] Wang J. (2016). 超越忠实执行与盲目抵制—教育改革中教师作为转化性知识分子的角色担当 [Beyond faithful implementation and blind resistance: teachers as transformative intellectuals in educational reform]. 中国教育学刊 [Chin. J. Educ.] 1, 78–83. Chinese.

[B109] Wang Z. Li K. Cheng F. (2007). 关于教师对新课程改革阻抗的思考 [Reflections on teachers' resistance to new curriculum reform]. 辽宁教育研究 [Liaoning Educ. Res.] 1, 57–59. Chinese.

[B110] Xia X. (2008). 教师抵制课程变革: 从变革的“障碍”到“动力 [Teachers' resistance to curriculum change: from obstacle to driving force]. 当代教育科学 [Contemp. Educ. Sci.] 2, 16–19. Chinese.

[B111] Yin X. (2015). 中小学教师对U–S合作培养师范生的阻抗分析–以现象学为视角 [An analysis of primary and secondary teachers' resistance to U–S collaborative teacher training: a phenomenological perspective]. 中国教育学刊 [Chin. J. Educ.] 5, 76–80. Chinese.

[B112] Zaltman G. Duncan R. (1977). Strategies for Planned Change. New York, NY: Wiley-Interscience.

[B113] Zhang Y. Wang J. Akhtar M. N. Wang Y. (2022). Authoritarian leadership and cyberloafing: a moderated mediation model of emotional exhaustion and power distance orientation. Front. Psychol. 13:1010845. doi: 10.3389/fpsyg.2022.1010845 36267076 PMC9577504

[B114] Zheng X. Shi X. Liu Y. (2020). Leading teachers' emotions like parents: relationships between paternalistic leadership, emotional labor and teacher commitment in China. Front. Psychol. 11:519. doi: 10.3389/fpsyg.2020.00519 32318001 PMC7147470

