# Supplementary material for: How paternalistic leadership influences teachers' resistance to STEM reform: the mediating role of teachers' STEM literacy and the moderating role of work engagement
Source: Front Psychol. 2026 Feb 23;17:1726148. doi: 10.3389/fpsyg.2026.1726148 (PMC12968017; doi:10.3389/fpsyg.2026.1726148)
Supplement: Supplementary file 1 [file Table_1.docx]

Supplementary Material

**How Paternalistic leadership Influences Teachers’ Resistance to STEM Reform: The Mediating Role of Teachers’ STEM Literacy and the Moderating Role of Work Engagement**

Yao Zhang, Dongchen Zhao

The supplementary material for this article includes the complete items lists of four scales used in this study. Items marked with an asterisk (*) require reverse coding while scoring.

**Teachers’ Resistance to STEM Scale**

The *Teachers’ Resistance to STEM Scale* consists of 3 dimensions and 11 items.

**Affective resistance**

1. I am afraid that STEM courses will change my established teaching practices.

2. I do not like learning about or implementing STEM courses.

3. I am afraid of losing control of the classroom when implementing STEM courses.

4. Implementing STEM courses makes me feel stressed.

**Cognitive resistance**

5. I think that implementing STEM courses will create difficulties for the school’s teaching.

6. I believe that implementing STEM courses is negative.

7. I believe that implementing STEM courses will benefit the school or students.*

**Behavioral resistance**

8. I protested against the implementation of STEM education in my school.

9. I complained to my colleagues about the implementation of STEM education.

10. I expressed my objections to school administrators regarding the implementation of STEM education.

11. I spoke positively about the implementation of STEM education to others.*

**Paternalistic Leadership Scale**

The *Paternalistic Leadership Scale* consists of 2 dimensions and 9 items.

**Benevolence–moral leadership**

1. The leader expresses concern about teachers’ private lives and daily living.

2. The leader meets teachers’ needs according to their requests.

3. The leader helps teachers resolve tough problems in their daily lives.

4. The leader treats teachers fairly without bias.

5. The leader is a role model for teachers in terms of moral character and performance.

**Authoritarian leadership**

6. Teachers feel pressured when working with the leader.

7. The leader scolds teachers when they cannot accomplish their tasks.

8. The leader does not share information with teachers.

9. The leader is aloof in the presence of teachers.

**Teachers’ STEM Literacy Scale**

The *Teachers’ STEM Literacy Scale* consists of 4 dimensions and 18 items.

**Value orientation toward STEM education**

1. I agree that STEM education is important in cultivating the nation's future talent.

2. I agree that STEM education is valuable for students' future development.

3. I can explore and develop the educational value of the disciplines I teach from a STEM perspective.

4. I agree that STEM education can enhance students' key competencies such as scientific literacy, innovative spirit, and practical abilities.

**Disciplinary foundations and interdisciplinary integration**

5. I possess the engineering design thinking required to conduct STEM education.

6. I possess the scientific inquiry literacy required to conduct STEM education.

7. I possess the technological literacy required to conduct STEM education (e.g., educational technology, information technology, computer programming).

8. I possess the mathematical literacy required to conduct STEM education.

9. I understand the basic characteristics of the disciplines: Science (S), Technology (T), Engineering (E), and Mathematics (M).

**Development and implementation of STEM curriculum**

10. I can design STEM courses with a student-centered approach.

11. I can enrich the teaching and learning by integrating content from multiple disciplines, e.g., Science (S), Technology (T), Engineering (E), and Mathematics (M).

12. When designing STEM courses, I can effectively utilize teaching resources both inside and outside the school.

13. When designing STEM courses, I consciously cultivate students' thinking skills (critical thinking, creative thinking, scientific thinking, computational thinking, and design thinking, etc.).

14. I can utilize various technologies and venues to create STEM spaces suitable for interdisciplinary learning.

**Curriculum evaluation**

15. I can use assessments to understand students' progress at each stage of STEM learning (starting point, process, and outcome).

16. I can comprehensively evaluate students' STEM literacy in all aspects.

17. I can use diverse assessment methods to measure students' STEM learning outcomes.

18. I can provide students with timely feedback on their STEM learning to help them improve their learning.

**Work Engagement Scale**

The *Work Engagement Scale* consists of 4 items. Scores can be calculated summed for an overall score.

1. At my job, I feel strong and vigorous.

2. I am enthusiastic about my job.

3. My job inspires me.

4. I feel happy when I am working intensely.
